# Retained Placenta and Postpartum Hemorrhage: A Case Report and Review of Literature

**DOI:** 10.7759/cureus.24389

**Published:** 2022-04-22

**Authors:** Lucie Sorelle Tchuinte Lekuikeu, Connie Moreland

**Affiliations:** 1 Obstetrics and Gynecology, Mount Sinai Hospital, Chicago, USA

**Keywords:** normal spontaneous vaginal delivery (nsvd), risk factors, cesarian delivery, uterotonics, placenta accreta spectrum, retained placenta

## Abstract

The third stage of labor (delivery of the placenta), per current definition, takes place within 30 minutes of fetal delivery in a nulliparous or multiparous woman. According to the American Pregnancy Association, a retained placenta is diagnosed if the placenta is not delivered within 30 minutes following delivery of the fetus. Retained placenta can be caused by placenta accreta, increta, or percreta. There are several complications of a retained placenta, including postpartum hemorrhage, which can lead to maternal death if not treated promptly. We report the case of a 32-year-old female, gravida 4 para 3, who was diagnosed with a retained placenta after delivering at term (39 weeks gestation). The retained placenta was complicated by postpartum hemorrhage and was treated within 15 minutes of fetal delivery with several uterotonics (misoprostol, oxytocin, carboprost, and tranexamic acid) and several passes of ultrasound-guided suction curettage. Sharp curettage was also used with ultrasound to confirm that the uterus was empty, followed by one more suction curettage to remove any products of conception that were scraped off with sharp curettage. Vaginal bleeding was significantly reduced; minor bleeding was noted from a first-degree vaginal laceration, which was repaired by suture. The patient recovered from surgery and was discharged on postpartum day 3 with her neonate in stable condition. In conclusion, this case highlights that retained placenta is a serious obstetric complication that can cause life-threatening postpartum hemorrhage. More data are needed to define the period of time correlating with the greatest chance of encountering a retained placenta in order to improve obstetric care and reduce maternal morbidity and mortality. Future research should consider challenging the current definition of retained placenta, defined as a placenta undelivered after 30 minutes, in favor of a shorter time period, 15 minutes undelivered, in order to mobilize the obstetric team, anesthesiologist, and blood bank to prevent catastrophic postpartum hemorrhage.

## Introduction

A retained placenta is clinically diagnosed during the third stage of labor when the placenta fails to deliver within 15-30 minutes after childbirth [1,2]. The most common reason why the placenta is retained after childbirth is that placental villi adhere to the uterine myometrium without invading the decidua (placenta accreta), or the villi do invade either the myometrium (increta) or the uterine serosa and adhere to various nearby organs like the bladder (percreta). The incidence of the placenta accreta spectrum (accreta, increta, percreta) has increased from 1 in 4,017 in the 1980s to about 1 in 533 from 1982 to 2002, and about 1 in 272 for women who had a birth-related hospital discharge diagnosis in 2016 [3,4]. This increase is due to a corresponding increase in cesarean deliveries [5]. Other risk factors of the placenta accreta spectrum (previously known as morbidly adherent placenta) include a previous history of the adherent placenta, multiple gestations, placenta previa diagnosed during pregnancy, multiparity, advanced maternal age, and Asherman syndrome [6]. Although most placenta previa diagnosed in the first trimester may resolve by the third trimester, persistent previa or newly diagnosed placenta previa in the third trimester of pregnancy increases the risk of placenta accreta spectrum in the current or future pregnancy. Retained placenta can be benign (removed via manual extraction or curettage, as in our case report). However, it can be life-threatening when the placenta is morbidly adherent to the uterus. Morbidity and mortality from adherent placenta and subsequent postpartum hemorrhage are higher in underdeveloped countries and in hospitals without adequate medical equipment to handle high-risk cases. The retained placenta has a case mortality rate of nearly 10% in rural areas [7] and should be diagnosed and treated early to avoid potentially fatal complications. It is therefore important to continuously research ways to adequately treat retained placenta and to review existing literature on the current treatment used in various healthcare settings. We hereby report a case of retained placenta diagnosed in the third stage of labor and treated with several uterotonics, including misoprostol, oxytocin (Pitocin), carboprost (Hemabate), tranexamic acid, and ultrasound-guided suction curettage.

## Case presentation

A 32-year-old female, gravida 4 para 3, presented at 39 weeks and 4 days (dated by last menstrual period and first-trimester ultrasound) for induction of labor. She had three previous normal spontaneous vaginal deliveries (NSVD) and was diagnosed with chronic hypertension during this pregnancy. The patient denied any history of abortion, miscarriage, or any other medical history, including complications in previous pregnancies or deliveries. The patient also denied any previous surgeries. She did not use tobacco, nicotine, alcohol, opiates, or other illicit drugs during this pregnancy. She denied abdominal pain, leakage of fluids, and vaginal bleeding. She admitted to being treated for chlamydia in the remote past. She screened negative for sexually transmitted diseases (human immunodeficiency virus [HIV], herpes simplex virus [HSV], syphilis, chlamydia, gonorrhea, trichomoniasis) during her first and third trimesters. She screened positive for group B streptococcus (GBS) in the third trimester. A review of the systems was negative. She endorsed the good fetal movement, and the non-stress test was reassuring. The patient was admitted to labor and delivery for induction of labor due to chronic hypertension and was started on penicillin for GBS treatment.

Course of labor

Labor was induced with misoprostol for cervical ripening, foley balloon placement for mechanical dilation, and IV (intravenous) oxytocin for stimulation of contractions. She received epidural anesthesia. The patient had a normal labor curve, and after 13 hours of labor, she delivered via normal spontaneous vaginal delivery. After cutting the umbilical cord and obtaining a cord sample for arterial and venous blood gases, gentle traction was maintained for 15 minutes to deliver the placenta; however, the placenta did not deliver. Following delivery, IV oxytocin was given continuously to the patient. The bladder was noted to be full and thus was emptied; approximately 200 mL of clear urine was drained. Traction was maintained for an additional 15 minutes, after which the placenta was noted to have only partially separated from the uterine fundus. Moderate bleeding was noted; hence, an attempt was made to manually deliver the placenta after the administration of 4 mg of morphine. Upon manual extraction, only two-thirds of the placenta was removed, with the remaining third still adhered to the fundus. Given the risk of severe hemorrhage, the patient properly consented to a suction curettage with a possible hysterectomy. She also verbalized consent for the possible transfusion of blood products.

Operating room course

After consent, the patient was taken urgently to the operating room. She was prepped for a suction curettage under ultrasound guidance and general anesthesia was administered. After preparing the patient for curettage, a 12 French curved suction curette was inserted gently into the fundus. The suction machine was turned on and multiple passes of suction curette were used with ultrasound guidance to remove the retained products of conception. Sharp curettage was then used (with ultrasound) to confirm that the uterus was empty, followed by one more suction curettage to remove any products of conception that were scraped off with the sharp curette. The patient received 800 mcg of rectal misoprostol, 50 units of IV oxytocin, 250 mcg of subcutaneous carboprost, and two IV doses of 1 g of tranexamic acid intra-operatively to control postpartum hemorrhage. Vaginal bleeding was significantly reduced after clearing the uterus from the placenta and products of conception and administering numerous intravenous uterotonics. Minor bleeding was noted from a first-degree vaginal laceration, which was repaired with a 3-0 vicryl suture. Upon repair of the vaginal laceration, the bleeding stopped completely, and the procedure was concluded. The patient lost 1667 mL of blood and received 3 units of packed red blood cells (pRBCs), 1 unit of fresh frozen plasma (FFP), and 1 unit of platelets. The various portions of placenta collected from this patient were sent to pathology for analysis. The patient was awakened from anesthesia and taken to the recovery room, awake, alert, and in stable condition.

Postoperative course

The patient was stable with minimal vaginal bleeding or hemorrhage during the next two days post-surgery. Her uterine fundus was firm and non-tender. She had a minor complaint of back pain, which she mentioned having before pregnancy and possibly being exacerbated by epidural anesthesia. Her back pain was controlled with non-steroidal anti-inflammatory medication. Although the patient previously denied (upon admission) a history of complications with her three NSVDs, she was later admitted (on postpartum day 1) to having one previous episode of postpartum hemorrhage secondary to retained placenta. The patient was discharged on the third day with her neonate, both in stable condition.

Pathology report

**Figure 1:**
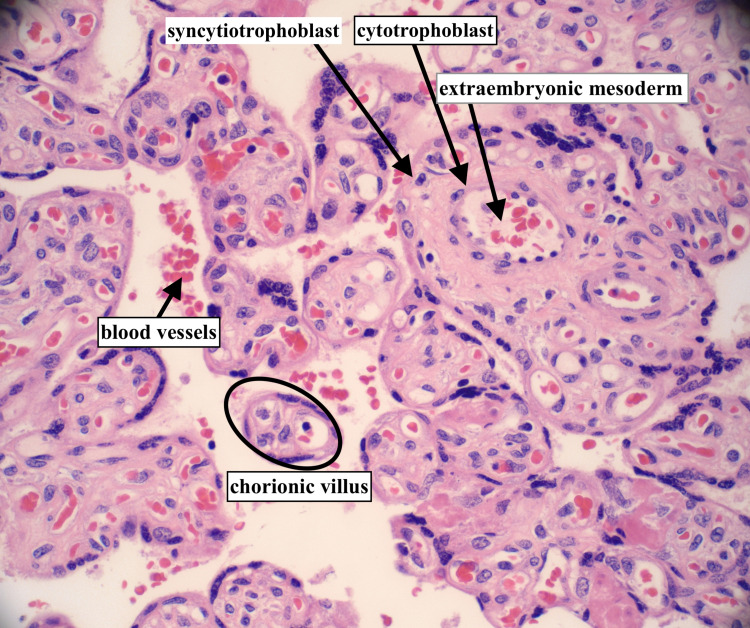
Normal placenta villi Normal placenta/chorionic villi with fetal blood vessels, syncytiotrophoblast, cytotrophoblasts and extra-embryonic mesoderm (hematoxylin and eosin stain, 40× magnification)

**Figure 2:**
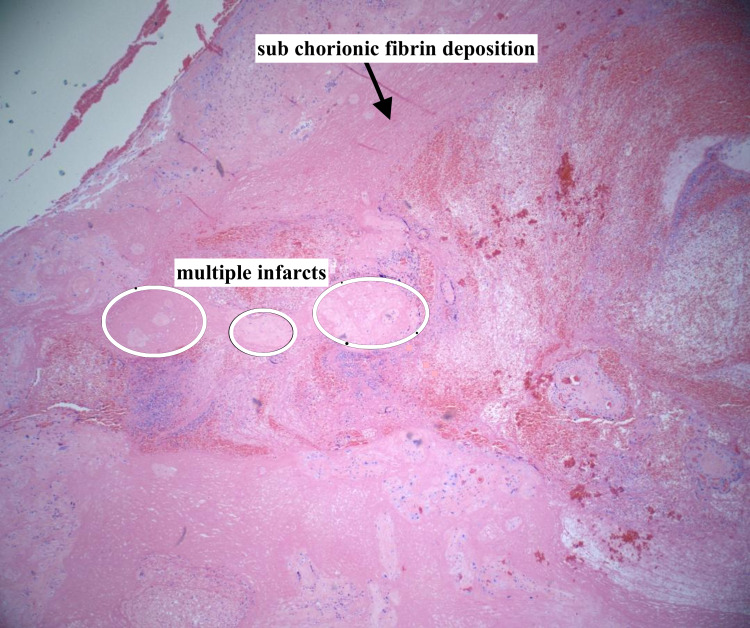
Multiple placental infarcts and sub chorionic fibrin deposition Placental infarcts comprised <10% of the placental volume (hematoxylin and eosin stain, 4× magnification)

**Figure 3:**
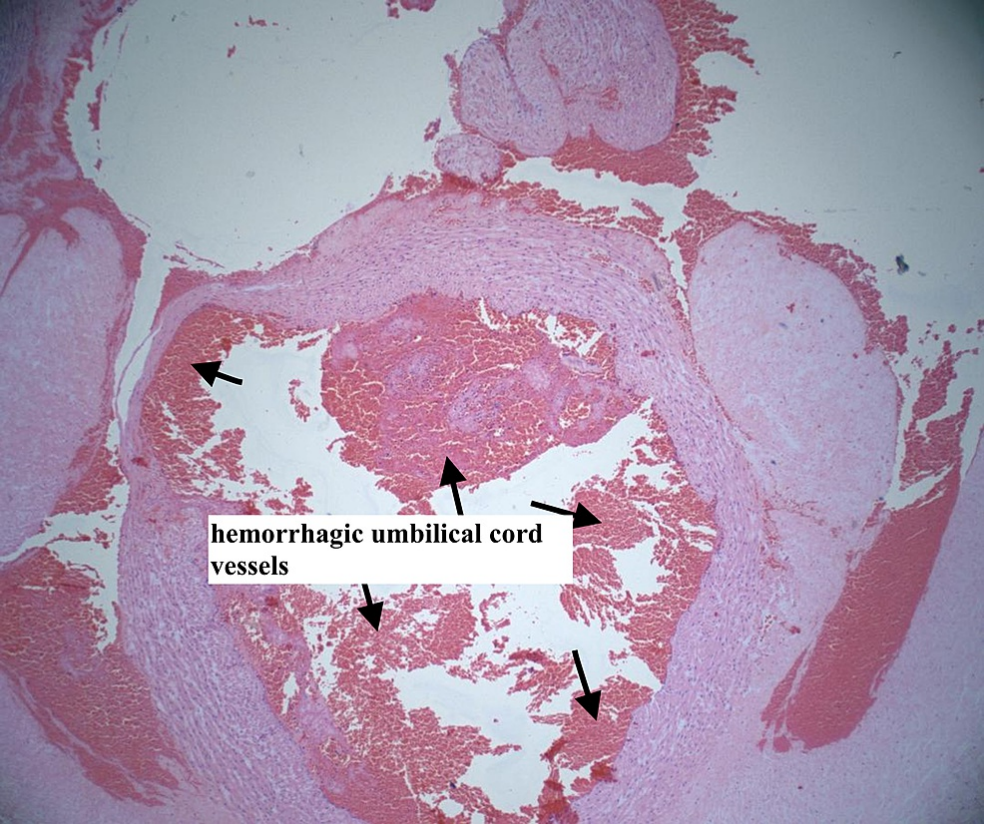
Hemorrhagic umbilical cord vessels Hemorrhagic umbilical cord vessels due to traction (hematoxylin and eosin stain, 4× magnification)

The patient’s placenta was analyzed, and the pathology report showed two specimens of placenta weighing 202 g and 188 g after the removal of the cord and membranes. The specimens comprised the torn, partial placenta, occasional normal placenta villi (Figure 1), some retained fragments, multiple small infarcts comprising less than 10% of the placental volume, and subchorionic fibrin deposition (Figure 2). The fetal membranes did not have any pathological features. There were also areas of umbilical vessel hemorrhage, likely due to umbilical cord traction during attempts to deliver the placenta (Figure 3). A diagnosis of the morbidly adherent placenta (specifically placenta accreta or increta) could not be made because the placenta was delivered in several pieces and it was not possible to find the adherence point of the placenta to the fundus during microscopic analysis. As such, the final diagnosis was retained placenta with multiple small infarcts less than 10% of placental volume (Figure 2).

## Discussion

This case highlights the presentation of the retained placenta, which can be asymptomatic throughout pregnancy and delivery and can only be diagnosed postpartum. The retained placenta may not show any ultrasound findings of placenta previa or accreta spectrum. As such, although it is important to develop protocols to manage known cases of the adherent placenta, clinicians must remain cognizant of the possibility of a retained placenta in a patient with little or no risk factors and should be ready to use their best clinical judgment if such a case is encountered. Inquiring about previous complications in labor and delivery can guide the clinicians and possibly prepare them to manage a suspected case of retained placenta. Our patient denied complications during her three prior NSVD, but postpartum admitted to having a previous episode of retained placenta. Prior history of the adherent placenta and prior dilation and curettage (which our patient may have had to manage her previous adherent placenta) increased the risk of our patient developing a retained placenta in a future pregnancy. Other risk factors include endometrial scarring due to endometritis, maternal age of more than 35 years, multiparity, submucous fibroids, and deposition of the embryo close to the cervix during embryo transfer with assisted reproductive technology [8].

We believe our patient likely had a focal placenta accreta, causing a retained placenta that could only be retrieved (in several pieces) after multiple passes of suction curettage. One potential risk of suction and curettage could be the removal of healthy uterine tissue and resultant uterine adhesions (if curettage is used excessively), possibly leading to Asherman syndrome. In contrast, leaving healthy tissue attached to the retained placenta could lead to acute hemorrhage, shock, and even death.

This case also adds to the existing literature about the early and stepwise management of retained placenta, showing that bleeding can be stopped without necessarily performing a hysterectomy (especially if the morbidly adherent placenta was not diagnosed during pregnancy and the patient desires future fertility). A 2005 prospective observational study by Magann et al. assessed postpartum hemorrhage in women with vaginal deliveries [9]. "Using receiver operating characteristic curves, the authors showed that 95% of normal placental delivery occurs within 18 minutes and that the third stage of labor lasting longer than 18 minutes is associated with a significant risk of postpartum hemorrhage" [10]. A 2012 follow-up study by Magann et al. included a randomized controlled trial assigning vaginal deliveries to manual removal at either 10 or 15 minutes (as opposed to the traditional 30 minutes) of the undelivered placenta [11]. The authors found that removal of an undelivered placenta at 15 minutes had a significantly greater likelihood of hemorrhage compared to removal at 10 minutes [11], thereby advocating for earlier intervention in the management of retained placenta.

Treatment of our patient's retained placenta and postpartum hemorrhage was initiated within 15 minutes of fetal delivery and undelivered placenta with several uterotonics (misoprostol, oxytocin, carboprost, and tranexamic acid) and several passes of ultrasound-guided suction curettage. Similar protocols have been established and used to manage morbidly adherent or retained placentas. For instance, a recent (2021) case report from AlMousa et al. [1] explains the use of ultrasound-guided curettage to remove the retained placenta. AlMousa et al. [1] also utilized and recommended additional imaging after the curettage (including pelvic magnetic resonance imaging [MRI]) to ensure that there was no more retained placenta (which could serve as a nidus for infection or pose an acute hemorrhage risk) prior to discharging the stable patient. Other management techniques currently used for retained placenta include intra-cervical nitroglycerin [12], intra-umbilical injection of oxytocin (into an umbilical vein) [13], carbetocin, and other uterotonics. The use of some of these techniques is controversial. In 2011, a Cochrane Review study assessed the use of umbilical vein oxytocin either alone or with intravenous oxytocin to reduce the need for manual removal of the retained placenta [14]. Although umbilical vein oxytocin is inexpensive and easily done, all well-designed randomized control trials showed no significant effect of umbilical vein oxytocin on reducing the need for manual removal of the retained placenta [14]. Last but not least, management involves the continuous use of uterine massage along with uterotonics. Even after treatment of retained placenta with resultant postpartum hemorrhage, it is important to express any residual bleeding via uterine massage to prevent further hemorrhage postpartum.

It is worth noting that uterotonics have contraindications. For example, methergine is contraindicated in hypertension or vasospastic diseases (like Raynaud’s phenomenon) and hemabate, a synthetic prostaglandin, is contraindicated in asthma, active hepatic, or renal disease. Our patient did not receive methergine due to her contraindication (chronic hypertension). She had no history of asthma, active hepatic disease, or renal failure; therefore, hemabate was appropriately given intra-operatively to control her bleeding.

Furthermore, it is imperative to make the right clinical judgment early in a case of postpartum hemorrhage secondary to retained placenta. If placenta accreta is clearly diagnosed on ultrasound (usually by visualizing placental septations deep into the uterus and/or nearby organs), a hysterectomy with the placenta in situ is often the best course of action to decrease maternal and fetal mortality, as attempts to manually extract such a morbidly adherent placenta are usually unsuccessful. The longer clinicians wait before performing a life-saving hysterectomy with the placenta in situ in such a patient, the higher the risk of maternal mortality due to uncontrolled hemorrhage and disseminated intravascular coagulation.

## Conclusions

Retained placenta can occur without identifiable antepartum risk factors. The incidence of the retained placenta has increased during the last few decades due to a corresponding increase in cesarean deliveries and other risk factors in the placenta accreta spectrum. Retained placenta can cause postpartum hemorrhage, which can be fatal. Careful history-taking, physical examination, and clinical judgment should be taken into consideration when suspecting and diagnosing retained placenta. It is important to diagnose this pathology early in order to guide management, which may involve a life-saving hysterectomy. Certain available medical treatments for retained placenta are controversial, like the use of intra-umbilical oxytocin and intra-cervical nitroglycerin. More research is needed to improve the medical and surgical management of retained placenta, carefully taking into account a patient’s pre-existing medical conditions.

In conclusion, this case highlights that retained placenta is a serious obstetric complication that can cause life-threatening postpartum hemorrhage. More data are needed to define the period of time correlating with the greatest chance of encountering a retained placenta in order to improve obstetric care and reduce maternal morbidity and mortality. Future research should consider challenging the current definition of retained placenta, defined as a placenta undelivered after 30 minutes, in favor of a shorter time period, 15 minutes undelivered, in order to mobilize the obstetric team, anesthesiologist, and blood bank to prevent catastrophic postpartum hemorrhage.
